# Mouse embryonic stem cell‐derived motor neurons are susceptible to ferroptosis

**DOI:** 10.1002/2211-5463.13545

**Published:** 2023-01-24

**Authors:** Alejandra M. Martinez, Ahryun Kim, Cristina Aguilar Flores, Daoud F. Rahman, Wan Seok Yang

**Affiliations:** ^1^ Department of Biological Sciences St. John's University New York City New York USA

**Keywords:** ALS, calcium, ferroptosis, glutathione peroxidase 4, neuron, stem cells

## Abstract

Ferroptosis is a regulated form of cell death driven by the lethal accumulation of lipid peroxides in cell membranes. Several regulators of ferroptosis have been identified using cancer cell lines. However, the cellular pathways of ferroptosis in neurons remain poorly characterized. In this study, we used a mouse embryonic stem cell‐derived motor neuron model to investigate how motor neurons respond to ferroptosis inducers. Pharmacological and genetic inhibition of glutathione peroxidase 4 (GPx4) induced ferroptosis in motor neurons, while system x_c_^−^ inhibition by erastin had no effect. RNA‐seq analysis showed that the expression levels of several genes were altered during RSL3‐induced ferroptosis. Subsequent bioinformatic analysis revealed alterations in several biological pathways during ferroptosis, including synaptogenesis and calcium signaling. Finally, we found that edaravone, an FDA‐approved drug for treating amyotrophic lateral sclerosis (ALS) disease, rescued motor neurons from RSL3‐induced ferroptosis. Our data highlight the crucial role of GPx4 in ferroptosis regulation and demonstrate that stem cell‐derived motor neuron culture is a valuable model to study ferroptosis at the single‐cell level in a neuronal context.

## Graphical abstract

Ferroptosis is a form of regulated cell death driven by lipid peroxides. Here, we used mouse stem cell‐derived motor neurons to investigate which cellular pathways are altered during ferroptosis in motor neurons. We identified several biological pathways that were altered in motor neurons during RSL3‐induced ferroptosis. The results demonstrate the usefulness of the stem cell‐based model in studying neuronal ferroptosis.

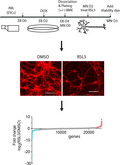

**Abbreviations**
ALS: amyotrophic lateral sclerosis
EBs: embryoid bodies
GPx4: glutathione peroxidase 4
GSH: reduced glutathione
iPSC‐MN: human‐induced pluripotent stem cell‐derived motor neuron
mES‐MN: mouse embryonic stem cell‐derived motor neuron
PUFAs: polyunsaturated fatty acids
SOD1: superoxide dismutase 1

Ferroptosis is a form of regulated cell death driven by the lethal accumulation of lipid peroxides in cell membranes [1]. It is distinct from other known cell death modalities such as apoptosis or necroptosis at the genetic, biochemical, and functional levels. One way to initiate ferroptosis is by inhibiting glutathione peroxidase 4 (GPx4), a major antioxidant enzyme that reduces lipid peroxides into lipid alcohols in cellular membranes. For example, the small molecule RSL3 binds to and inhibits GPx4 in cells and induces ferroptosis in many sensitive cancer cell lines [2]. Another way to induce ferroptosis is by inhibiting system x_c_^−^, a cystine‐glutamate antiporter, using the small molecule erastin [3]. Inhibition of system x_c_^−^ prevents cellular uptake of cystine, an oxidized di‐cysteine, and decreases the cellular cysteine pool, which leads to the depletion of cellular glutathione, a major antioxidant metabolite. Glutathione acts as a cofactor for many enzymes, including GPx4; therefore, blocking system x_c_^−^ inhibits GPx4 indirectly. Since the discovery of this key molecular mechanism, many other regulators of ferroptosis have been reported in various biological contexts [4]. These regulators carry out biological functions in diverse areas such as iron metabolism, amino acid metabolism, lipid metabolism, tumor suppression, and immune response. The growing list of ferroptosis regulators suggests that ferroptosis has a broad role in biology rather than a limited role applicable only in certain peculiar conditions.

Neurons are enriched with polyunsaturated fatty acids (PUFAs) so it is likely that neurons are sensitive to ferroptotic stimuli. The involvement of ferroptosis in neuronal cell death has been suggested in several experimental models. For example, HT22, a hippocampal cell line, underwent an oxidative form of cell death upon depletion of GSH, which shared many features of ferroptosis [5]. Knockout of the Gpx4 gene in mice was lethal and displayed prominent cell death in motor neurons [6]. Deuterated‐PUFAs, a specific inhibitor of ferroptosis [7], reduced brain damage in mouse models of Parkinson's disease [8] and Alzheimer's disease [9]. These data suggest that the ferroptosis pathway should be explored in the neuronal context to understand the diverse mechanisms of neuronal cell death and develop an alternative strategy for treating neurodegenerative diseases.

Stem cell‐derived neurons present a physiologically relevant model in studying the function of a neuron at the single‐cell level while requiring less time and resources than animal models [10]. As stem cell‐derived neurons are easily monitorable and controllable, they have quickly become the choice of an experimental platform for studying cellular mechanisms and performing screening projects. On the contrary, the stem cell‐derived neuron model has its limitations and challenges, such as the presence of immature neurons in the culture and the inability to provide a comprehensive view involving interaction with glial cells. Therefore, data from the stem cell‐derived neurons should be interpreted carefully and, ideally, be cross‐validated in other established models.

In this study, we used an engineered mouse embryonic stem cell‐derived motor neuron (mES‐MN) culture and examined ferroptosis induced by inhibition of GPx4. The strength of the mES‐MN model combined with next‐generation sequencing technology revealed several insights into the mechanism of ferroptosis in the motor neuron context.

## Results

We used an engineered mouse embryonic stem cell line, named iNIL [11], to produce motor neurons in a cell culture system. The genome of iNIL stem cells was engineered to contain an inducible expression cassette that drives the expression of three transcription factors, Ngn2, Isl1, and Lhx3, upon doxycycline treatment [11]. These transcription factors program iNIL stem cells to take spinal motor neuron fate, driving the stem cells' differentiation into spinal motor neurons with the help of neurotrophic factors supplied by a differentiation medium. In addition, iNIL cells harbor a GFP transgene whose expression is driven by the Hb9 promotor, which allows for quantitative analysis of motor neuron production.

**Fig. 1:**
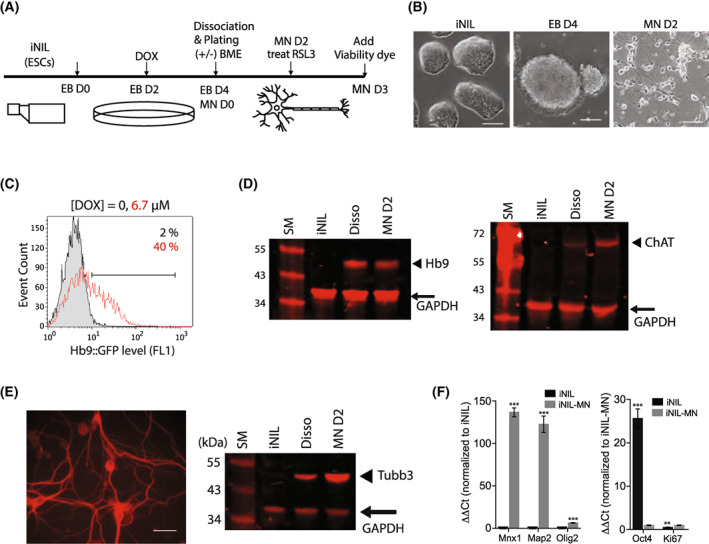
Differentiation of iNIL stem cells into motor neurons. (A) A time line diagram of the differentiation protocol. Starting from a frozen stock of iNIL, it takes 10 days until we get alamar blue viability data. (B) Representative images of iNIL stem cells, embryo bodies, and motor neurons. The white bar indicates 100 μm. (C) Flow cytometric determination of Hb9::GFP‐positive cell population from iNIL‐MN culture. (D) Western blot shows the expression of motor neuron markers (Hb9 and ChAT) upon differentiation of iNIL cells. Arrowheads indicate the Hb9 band (left) and ChAT (right), respectively. (E) Immunocytochemistry with class III beta‐tubulin antibody (left) and western blot (right) further demonstrate the motor neuron character of iNIL‐MNs. The white bar indicates 30 μm. (F) iNIL‐MN culture was further characterized by examining gene expressions of the indicated makers using RT‐qPCR analysis. Cells were harvested from either iNIL stem cell culture (iNIL) or motor neuron day 2 culture (iNIL‐MN). Data were presented as mean ± SEM; *n* = 3; ***P < 0.001, **P < 0.01, Student's *t*‐test. Data were technical replicates from a single differentiation batch.

iNIL cells were cultured on a flask and transferred to a low attachment dish to form embryoid bodies (EBs). Differentiation to motor neurons was started by adding doxycycline to the EB culture. After 2 days of induction, single cells were dissociated from the EB and plated on poly‐l‐ornithine‐coated culture vessels [11] (Fig. 1A). Two days after plating, the differentiated cells displayed neuronal morphology with long neurites extending from the cell bodies (Fig. 1B). FACS analysis quantification indicated that 40% of the culture at the time of dissociation were Hb9::GFP‐positive early motor neurons (Fig. 1C).

We performed western blot analysis using cell lysates obtained from the differentiated iNIL motor neuron (iNIL‐MN) cells at several time points to confirm the protein expression of motor neuron markers. We found that endogenous Hb9 expression level was increased up to 2 days after the plating step in response to a combination treatment of doxycycline induction and neurotrophic factors (Fig. 1D, left). Choline acetyltransferase (ChAT) is an enzyme required to synthesize the neurotransmitter acetylcholine. The expression of ChAT was upregulated throughout the time frame we examined (Fig. 1D, right). Class III β‐tubulin, a member of the tubulin family found exclusively in neurons, was also upregulated throughout the time frame (Fig. 1E, right). We used an antibody against class III β‐tubulin to perform immunocytochemistry and visualized motor neuron networks formed by iNIL‐MN cells (Fig. 1E, left). To further characterize iNIL‐MN culture created by our approach, we performed a real‐time quantitative PCR experiment and analyzed the gene expression levels of additional markers for stem cells and motor neurons. The markers included Oct4 (stem cell marker), Mnx1 (early motor neuron marker; Hb9), Map2 (neuronal marker), Olig2 (oligodendrocyte marker), and Mki67 (proliferation marker). The differentiated iNIL‐MNs expressed Mnx1 and Map2 more than 100‐folds compared with iNIL stem cells, which demonstrated strong enrichment of motor neurons in our iNIL‐MN culture (Fig. 1F, left). Olig2 upregulation was also detected in NIL‐MN culture, albeit to a much lesser degree (sixfold increase compared with iNIL stem cells), which suggested that our iNIL‐MN culture contained a small number of oligodendrocytes (Fig. 1F, left). By contrast, iNIL stem cells expressed 25‐fold higher level of Oc4 (stem cell marker) compared with iNIL‐MNs (Fig. 1F, right). Interestingly, we observed a slight increase in Ki67 expression (proliferation marker, 1.8‐fold increase compared with iNIL stem cells), which suggested the existence of undifferentiated, proliferating cells in our iNIL‐MN culture (Fig. 1F, right). Overall, these data showed that the doxycycline‐inducible system successfully created motor neurons from the iNIL stem cells.

**Fig. 2:**
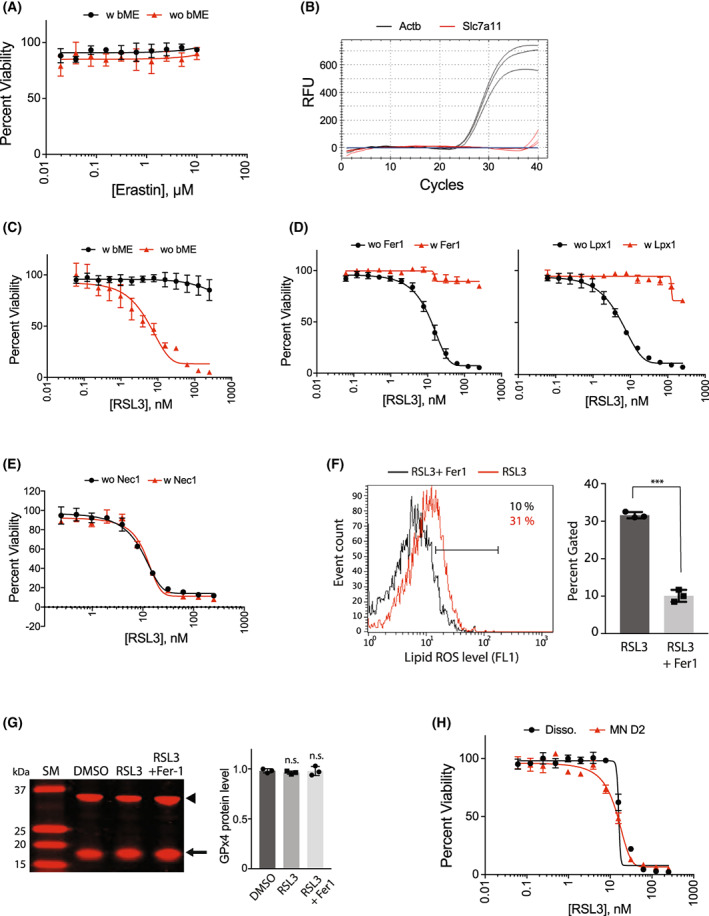
RSL3 but not erastin‐induced ferroptosis in iNIL‐MNs. (A) Erastin did not induce cell death in iNIL‐MNs. Erastin was treated for 24 h. (B) Quantitative reverse transcription PCR (RT‐PCR) showed iNIL‐MNs did not express Slc7a11, the target protein of erastin. (C) iNIL‐MN was sensitive to RSL3 treatment. Cells were treated with RSL3 for 24 h. (D) Fer‐1 (1 μm) and Lpx‐1 (1 μm) rescued cells from RSL3‐induced ferroptosis. (E) Nec‐1 (10 μm), a necroptosis inhibitor, could not rescue cells from RSL3‐induced ferroptosis. (F) Monitoring lipid‐ROS level using BODIPY‐C11 dye. RSL3 (10 nm) treated iNIL‐MNs displayed a higher lipid‐ROS level than a control sample co‐treated with Fer‐1 (1 μm). Data are presented as mean ± SD, *n* = 3, ***P < 0.001, Student's *t*‐test. Data points are technical replicates from a single differentiation batch. (G) RSL3 treatment did not reduce GPx4 protein level. Arrow indicates GPx4 band, and the arrowhead indicates GAPDH band as a loading control. Data were presented as mean ± SD, *n* = 3, n.s., not significant, Student's *t*‐test. Data points are biological replicates from three independent differentiation batches. (H) Motor neuron maturation did not change cells' sensitivity against RSL3. RSL3 was treated to either dissociated cells or motor neurons matured for 2 days. Data in the dose response curve were presented as mean ± SD; *n* = 3. The viability data were technical replicates from a single differentiation batch from the iNIL stem cells.

Seeing that there is no existing report on the involvement of ferroptosis in mouse stem cell‐derived motor neurons, we first tested erastin, a system x_c_^−^ inhibitor [1, 3] to see whether it can induce ferroptosis in the iNIL‐MNs. Note that we used B27 media without antioxidants throughout the experiments because the antioxidants might interfere with ferroptosis assays. As shown in Fig. 2A, erastin treatment did not affect the survival of iNIL‐MNs that were grown under a standard motor neuron media containing beta‐mercaptoethanol (bME). The bME in the media acts as a reducing agent that converts a cystine molecule into two molecules of cysteine. Then, the reduced cysteines are imported into the cells through the action of membrane amino acid transporters that is different from system x_c_^−^ [3]. Therefore, cells can uptake cysteine, a precursor of glutathione, regardless of the system x_c_^−^ inhibition by erastin. This result suggested that we should remove bME from the culture media to see erastin's effect. However, erastin did not change iNIL‐MNs' viability even after the removal of bME from the culture media (Fig. 2A). These results suggested that iNIL‐MNs are inherently resistant to erastin due to an intrinsic factor. *Slc7a11* is a gene encoding a regulatory subunit of system x_c_^−^, and its expression is required for a functional system x_c_^−^. As the expression pattern of *Slc7a11* varies widely among different cell types, we performed a real‐time PCR analysis to determine the mRNA level of *Slc7a11* and found that iNIL‐MNs did not express *Slc7a11* (Fig. 2B). This explains why erastin was not lethal to iNIL‐MNs, regardless of bME, because there was no target protein in the iNIL‐MNs.

Next, we tested (1S,3R)‐RSL3 (RSL3 hereafter), a direct inhibitor of GPx4 enzyme [2, 7], to see whether it can induce ferroptosis in iNIL‐MNs. GPx4 is an antioxidant enzyme that uses GSH as a cofactor and reduces lipid peroxides to lipid alcohol. Inhibition of GPx4 causes lethal accumulation of lipid peroxides in cell membranes leading to ferroptotic cell death. Unlike erastin, RSL3 treatment killed iNIL‐MNs with high potency (EC_50_ = 10 nm) when bME was removed from the culture media (Fig. 2C). By contrast, the addition of bME protected iNIL‐MNs from RSL3's lethality. The bME is known to have a radical scavenging activity [12]. As RSL3's lethality is independent of system x_c_^−^, this result suggests that the radical scavenging activity of bME, rather than the reduction in cystine to cysteine, rescued iNIL‐MNs from cell death.

When RSL3 was treated in the presence of ferrostatin‐1 (Fer‐1) or liproxstatin‐1 (Lpx‐1), the two ferroptosis specific inhibitors [13, 14], cells were rescued, which confirmed that iNIL‐MNs were dying through ferroptosis (Fig. 2D). In comparison, Nec‐1, a necroptosis inhibitor, could not rescue cells from RSL3‐induced cell death (Fig. 2E). A biochemical marker for ferroptosis is the generation of lipid‐peroxides that disrupt the cell membrane. We used BODIPY‐C11, a dye that emits green fluorescence upon exposure to lipid‐peroxides, to determine lipid‐peroxide levels in RSL3‐treated iNIL‐MNs using flow cytometry. Because the GFP signal from Hb9::GFP in iNIL cells interferes with the BODIPY‐C11 signal, we used iNIL cells without Hb9::GFP (hereafter INF cells) for this assay. INF‐MNs treated with RSL3 had a higher level of lipid‐peroxides than control INF‐MNs co‐treated with Fer‐1 (Fig. 2F). Western blot analysis of iNIL‐MN cell lysates demonstrated the expression of endogenous GPx4, the target protein of RSL3 (Fig. 2G).

It was reported that RSL3 treatment to BJeLR and HT1080, the two cancer cell lines in which ferroptosis was originally identified, accompanied reduction in GPx4 protein abundance with unknown mechanisms [15, 16]. Interestingly, reduction in GPx4 protein still occurred even when cell death was completely rescued through co‐treatment with alpha‐tocopherol, a lipophilic antioxidant. The results suggested that the reduction in GPx4 protein was an event upstream of the lethal accumulation of lipid peroxides. We wondered whether RSL3‐mediated GPx4 protein reduction occurs in the motor neuron background and performed a western blot to monitor the GPx4 protein level in RSL3‐treated iNIL‐MNs. As shown in Fig. 2G, the GPx4 protein level remained stable during RSL3‐induced ferroptosis in iNIL‐MNs, showing that the mechanism of GPx4 protein reduction observed in the cancer cell lines did not work in our iNIL‐MN model.

Previously, we tested whether ferroptosis can be induced in NSC‐34, a motor neuron‐like cell line with limited motor neuron properties [17]. NSC‐34 is a hybrid cell line created by the fusion of mouse neuroblastoma and motor‐neuron enriched spinal cord cells [18]. Upon switching to a differentiation medium, the parental NSC‐34 cell changes its property to display more motor neuron‐like characters, such as an elongated neurite structure. We found that NSC‐34 cells under the differentiation condition were more sensitive to ferroptosis induction than the parental NSC‐34 cells [17]. To test whether sensitization to ferroptosis during motor neuron differentiation also occurs in our iNIL‐MN model, we compared RSL3 sensitivity between cells right after dissociation (MN D0, immature iNIL‐MNs) and cells that were grown for 2 days after dissociation (MN D2, more mature iNIL‐MNs). As shown in Fig. 2H, we did not see any difference in RSL3 sensitivity in either condition. It is likely that the reduced serum concentration in NSC‐34 differentiation media (1/10 of regular growth media) sensitized the differentiated NSC‐34 cells to ferroptosis due to the reduced amount of serum antioxidants in the media. By contrast, we used the same media for MN D0 cells and MN D2 cells in this experiment and did not see any difference in RSL3 sensitivity in iNIL‐MNs.

**Fig. 3:**
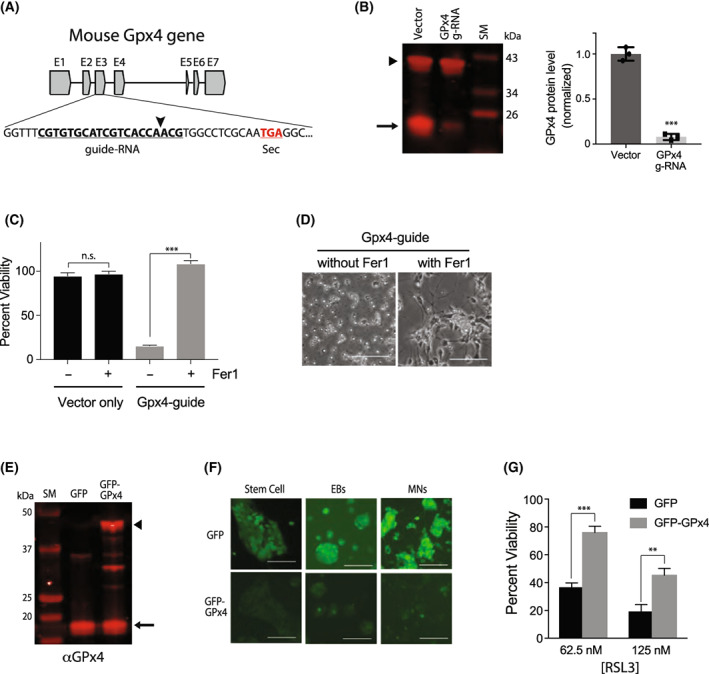
Genetic engineering of GPx4 modulated ferroptosis in INF‐MNs. (A) A diagram showing the structure of the mouse GPx4 gene and the target site for CRISPR gene knockout. (B) Western blot analysis showed decreased expression of GPx4 protein in INF‐MNs harboring GPx4‐guide RNA. Arrow indicates the GPx4 band, and the arrowhead indicates the GAPDH band. Data were presented as mean ± SD, *n* = 3, ***P < 0.001, Student's *t*‐test. Data points were technical replicates from a single differentiation batch for each cell line. (C) INF‐MNs with GPx4‐guide RNA died by ferroptosis. Cell viability was determined using alamar blue viability dye. Data were presented as mean + SD, *n* = 3, n.s., not significant, ***P < 0.001, Student's *t*‐test. Viability data were technical replicates from a single differentiation batch for each cell line. (D) Microscopic image of INF‐MNs expressing GPx4‐guide RNA in the presence or absence of Fer‐1 (1 μm). (E) Western blot analysis showing the protein expression of GFP‐GPx4. Arrow indicates endogenous GPx4 protein, and the arrowhead indicates GFP‐GPx4 fusion protein. (F) Live cell fluorescence microscope image of GFP and GFP‐GPx4 INF‐MNs at different stages of the differentiation process. GFP‐GPx4 showed a weaker signal than GFP throughout the differentiation. (G) INF‐MNs with GFP‐GPx4 survived better than cells with GFP. Cell viability was determined using alamar blue viability dye. The white bars in the microscope images indicate 30 μm. Data were presented as mean + SD, *n* = 3, ***P < 0.001, **P < 0.01, Student's *t*‐test. Viability data were technical replicates from a single differentiation batch for each cell line.

In cancer cell lines, knocking down GPx4 expression using RNAi induced ferroptotic cell death, which validated the mechanism of action for RSL3‐induced ferroptosis [2]. To confirm that RSL3 acts through the exact mechanism in our iNIL‐MN model, we reduced the expression of GPx4 using CRISPR technology and examined whether there were any changes in the viability of the cells. We designed guide‐RNA oligomers that target a site at exon3 of the mouse GPx4 gene (Fig. 3A). The site is located 14 nucleotides upstream of the catalytic selenocysteine codon. Therefore, the CRISPR‐mediated cut and subsequent end‐joining event through nonhomologous recombination will generate indel mutations that prevent the expression of full‐length GPx4 encoding the catalytic selenocysteine residue.

Lentiviruses harboring a GPx4‐guide RNA expressing plasmid were produced and infected to INF stem cells (INF = iNIL without Hb9::GFP reporter). The expression cassette of the plasmid contained a GFP reporter; therefore, we purified GPx4‐guide RNA expressing cells using a FACS machine. Note that we added 1 μm Fer‐1 to the stem cell growth media to maintain the culture because cell growth was significantly slower without the addition of Fer‐1. To confirm the effect of GPx4‐guide RNA, we prepared cell lysates. Cellular GPx4 protein was detected using antibodies against GPx4. As shown in Fig. 3B, INF stem cells expressing GPx4‐guide RNA contained significantly less GPx4 protein (91% less than vector‐only lysate; normalized to GAPDH band) than the control vector‐only lysate which showed an abundant GPx4 protein band. We then differentiated the INF stem cells into motor neurons as before and determined the cell viability in the presence or absence of Fer‐1. Motor neurons created from GPx4‐guide RNA engineered INF cells could not survive after dissociation (Fig. 3C,D left). By contrast, the addition of Fer‐1 to the motor neuron media rescued cells to the control level in which cells express a normal level of GPx4 (Fig. 3C,D right). These data indicate that INF‐MNs require an abundant amount of GPx4 for survival. The removal of toxic lipid peroxides by GPx4 may be essential for the survival of motor neurons. The data also demonstrate that genetic ablation of GPx4 phenocopies RSL3 treatment in our INF‐MN model.

Next, we wondered whether overexpression of GPx4 in INF‐MNs protects cells from RSL3‐induced cell death. INF stem cells were infected with lentiviruses harboring a GFP‐GPx4 cDNA expressing plasmid. GFP‐GPx4‐positive cells were purified using the FACS machine, and GFP‐GPx4 protein expression was determined using a western blot. The antibody against GPx4 demonstrated the expression of GFP‐GPx4 protein on the western blot image (Fig. 3E). However, when we observed GFP‐GPx4 in live INF stem cells, the GFP signal was significantly weaker than that of live GFP INF stem cells (Fig. 3F). The GFP signal from GFP‐GPx4 INF cells remained low throughout the cells' differentiation into motor neurons (Fig. 3F). Selenoprotein GPx4 requires special machinery to incorporate selenocysteine into an internal UGA codon that usually encodes STOP codon. This machinery consists of several cis and trans acting factors that include SECIS (selenocysteine insertion sequence), SBP2 (SECIS binding protein 2), and isoprenylated tRNA‐Sec. It is likely that the selenocysteine expression system in INF cells supports the endogenous expression of GPx4 but may need additional engineering in order to support overexpression of exogenous GPx4. For example, a research team was able to achieve the overexpression of exogenous GPx4 by coexpressing SBP2 in HEK cells [19]. Despite this technical issue, we went on testing RSL3 and found that INF‐MNs expressing GFP‐GPx4 were more resistant against RSL3 treatment than GFP‐expressing INF‐MNs (Fig. 3G). The data further corroborate the mechanism of RSL3‐induced ferroptosis in our motor neuron culture model.

**Fig. 4:**
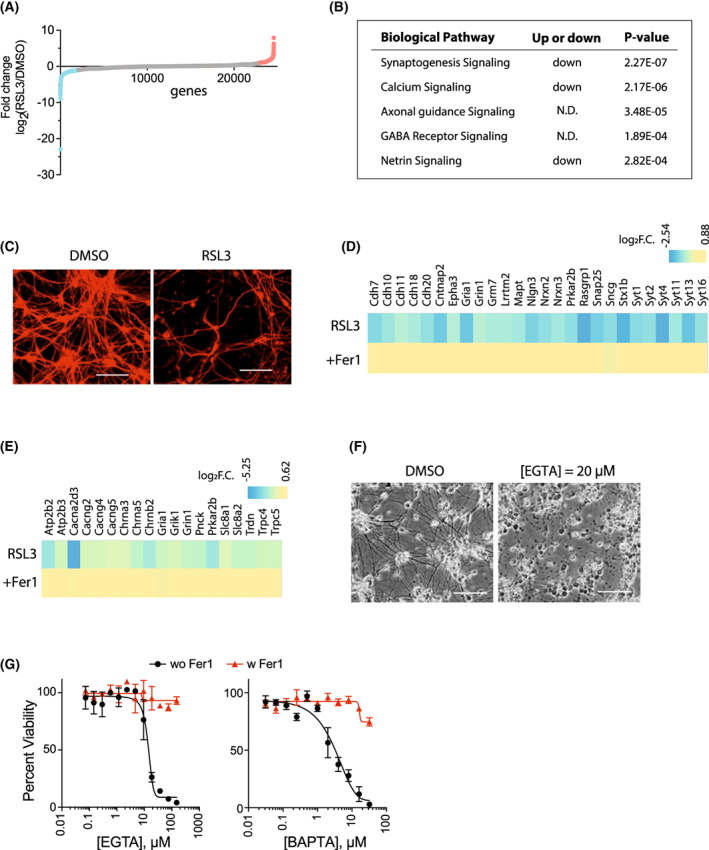
Gene expression profiling analysis revealed biological pathways altered during RSL3‐induced ferroptosis in iNIL‐MNs. (A) RNA‐seq analysis determined changes in the individual gene expression level in iNIL‐MN genome upon RSL3 treatment. Upregulated genes (greater than two folds) were marked as red, whereas downregulated genes (greater than two folds) were marked as blue. (B) IPA pathway analysis (Qiagen) identified biological pathways that were altered by RSL3‐treatment. (C) Immunocytochemistry with class III beta‐tubulin antibody showed much less neurite connections in RSL3‐treated iNIL‐MN culture, which supports downregulation of synaptogenesis signaling. The white bar indicates 30 μm. (D) The heatmap shows the names of genes that belong to the synaptogenesis signaling category and represents the fold changes in the expression level. Most of them are downregulated. (E) The heatmap shows the names of genes that belong to the calcium signaling category and represents the fold changes in the expression level. (F) iNIL‐MNs were dying by EGTA, a calcium chelator. Cells were treated for 24 h. (G) Fer‐1 rescued cell death induced by EGTA and BAPTA‐AM in iNIL‐MNs. One micromolar of Fer‐1 was used. Viability data in the graph were technical replicates from a single differentiation batch and presented as mean ± SD; *n* = 3.

Having confirmed that RSL3 could induce ferroptosis in iNIL‐MNs, we wondered what biological pathways downstream of GPx4 inhibition by RSL3 were modulated during ferroptosis in iNIL‐MNs. For this, we prepared mRNA samples from RSL3 treated and control iNIL‐MNs and performed RNA‐seq analysis to investigate changes in the gene expression levels upon ferroptosis induction. A total of 24 575 mouse genes were surveyed for expression changes. Individual genes showed varying degrees of upregulation and downregulation from RSL3 treatment (Fig. 4A and Tables S1 and S2 for full dataset). We required greater than twofold changes in the gene expression level and less than 0.05 in FDR adjusted *P*‐value (Benjamini–Hochberg; *n* = 5) in selecting 656 differentially expressed genes for further analysis.

Uploading the selected genes to Qiagen IPA (Ingenuity Pathway Analysis) tool identified several biological pathways that were altered by RSL3 treatment in iNIL‐MNs. The most significantly altered pathway was the ‘synaptogenesis signaling pathway,’ followed by the ‘calcium signaling pathway,’ and others (Fig. 4B). The synaptogenesis pathway becomes upregulated when the formation of synapses between neurons is promoted. As iNIL‐MNs die during RSL3‐induced ferroptosis, downregulation of the synaptogenesis signaling pathway is likely a reflection of the smaller number of synapses among motor neurons in RSL3‐treated culture (Fig. 4C). Suppression of ferroptosis by Fer‐1 restored the expression of synaptogenesis signaling pathway genes, which suggests that the downregulation of synaptogenesis genes occurred after lipid peroxide generation during RSL3‐induced ferroptosis (Fig. 4D).

Calcium signaling is involved in a variety of cellular processes, including neuronal cell death by glutamate‐induced excitotoxicity [20]. Our RNA‐seq analysis showed that many calcium channel genes (genes for voltage‐gated calcium channels, cholinergic receptors, and ionotropic glutamate receptors) were downregulated during RSL3‐induced ferroptosis in iNIL‐MNs, suggesting that cellular calcium level could be lowered by RSL3 treatment (Fig. 4E). To determine the role of calcium during the ferroptosis, we tried treating iNIL‐MNs with RSL3 in the presence of EGTA‐AM, a cell‐permeable calcium chelator. While setting up this experiment, we unexpectedly observed that EGTA‐AM alone was highly toxic to iNIL‐MNs, which prevented us from determining the effect of calcium chelation (Fig. 4F). Therefore, we investigated whether maintaining a normal calcium level was essential for iNIL‐MN survival. Testing EGTA‐AM in a twofold dilution series manner showed that an EGTA‐AM concentration as low as 20 μm was enough to reduce cell viability of iNIL‐MN to a significant level (Fig. 4G, left). Intriguingly, ferrostatin‐1 was able to rescue cells from death by EGTA‐AM treatment, indicating that calcium depletion by EGTA‐AM induced ferroptosis in iNIL‐MNs (Fig. 4G, left). We tested a second calcium chelator, BAPTA‐AM, and observed the similar pattern of ferroptosis induction through calcium depletion (Fig. 4G, right). It is not clear, however, how calcium depletion induced ferroptosis in the motor neuron context. In HT‐1080, a human fibrosarcoma cell line in which ferroptosis was originally characterized, there was no cytotoxicity by EGTA‐AM alone and no change in ferroptosis sensitivity under the co‐treatment condition [2]. On the contrary, depletion of luminal calcium protected mouse embryonic fibroblast cells from RSL3‐induced ferroptosis [21]. Therefore, it is likely that the effect of calcium on ferroptosis depends heavily on the cell type.

**Fig. 5:**
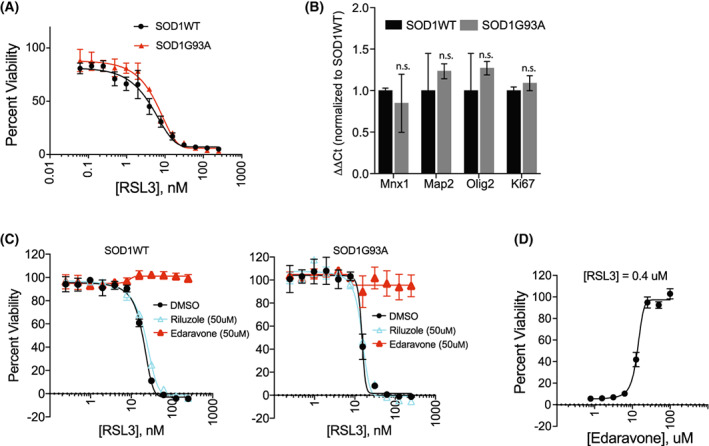
Edaravone, an ALS drug, suppressed RSL3‐induced ferroptosis in iNIL‐MNs. (A) iNIL cells expressing SOD1G93A did not change the cell's sensitivity against RSL3 treatment. Cells were treated for 24 h. (B) qPCR analysis showed that both cell lines behaved similarly during differentiation process. Data are presented as mean ± SEM; *n* = 3; n.s., not significant, Student's *t*‐test. Data points are technical replicates from a single differentiation batch for each cell line. (C) Edaravone but not riluzole rescued iNIL‐MNs with or without SOD1G93A mutation from RSL3‐induced ferroptosis. Fifty micromolar of riluzole or edaravone was treated with the indicated amount of RSzL3 for 24 h. (D) Determination of EC50 value of edaravone in inhibiting RSL3‐induced ferroptosis. Viability data were technical replicates from a single differentiation batch for each cell line and presented as mean ± SD; *n* = 3.

Stem cell‐derived motor neurons have been instrumental in modeling motor neuron diseases such as amyotrophic lateral sclerosis (ALS) [10]. Amyotrophic lateral sclerosis is a fatal paralytic disease caused by selective loss of motor neurons in the cerebral cortex and spinal cord. Human genetics studies have identified genetic loci, such as *C9ORF72*, *SOD1*, and *TDP‐43*, which harbor ALS‐causing mutations. Interestingly, postmortem spinal cord tissue samples from ALS patients contained lipid peroxidation products, which are hallmarks of ferroptotic cell death [22, 23]. Astrocyte conditioned media that express SOD1G93A generated reactive oxygen species in motor neurons and induced cell death [24]. These data led us to test whether ferroptosis is involved in motor neuron cell death seen in ALS disease. We grew isogenic iNIL stem cells with or without *SOD1G93A* mutation and produced motor neurons using our differentiation protocol. When we treated these cells with RSL3, they showed similar sensitivity toward RSL3‐induced ferroptosis with or without SOD1G93A (Fig. 5A). The two cell lines showed a similar pattern of differentiation marker profiles, which indicates that both cell lines behaved similarly during the differentiation (Fig. 5B). The results suggest that the cellular changes caused by *SOD1G93A* mutation in iNIL‐MN were not functionally connected to RSL3‐induced ferroptosis.

Currently, there are two major FDA‐approved drugs for treating ALS patients—edaravone and riluzole. We tested whether these two ALS drugs can protect iNIL‐SODWT and iNIL‐SODG93A motor neurons from RSL3‐induced ferroptosis. As shown in Fig. 5C,D, edaravone completely rescued both motor neurons from RSL3‐induced ferroptosis, whereas the effect of riluzole was neutral. The exact mechanism of how these drugs benefit ALS patients is unknown. However, edaravone is known to have an antioxidant activity [25], which may explain why it rescued iNIL‐MNs from RSL3‐induced ferroptosis. Since our stem cell‐derived motor neuron model is suitable for a high‐throughput, small molecule screening campaign, future effort to discover small molecule inhibitors of ferroptosis using our model may potentially help identify ALS drug candidates.

## Discussion

We used genetically engineered mouse embryonic stem cells and a transcriptional programming approach to produce motor neurons *in vitro*. The yield of the Hb9‐positive population was 40% at the time of dissociation, which was less than the previously reported yield [11]. Variations in the protocol at the level of detail and cellular heterogeneity are the possible cause of this difference. For example, the production of motor neurons from HBG3 stem cells yielded around 40% of Hb9::GFP‐positive motor neurons after induction with a series of small molecules and protein factors [26]. However, to achieve a similar level of Hb9::GFP positives from the same HBG3 cell line, another laboratory had to modify the original procedure, such as adding neurotrophic factors 2 days before the dissociation step [27]. If we want to remove the confounding effects from Hb9::GFP negative cells in the culture, we should use a flow cytometry machine to purify Hb9::GFP‐positive cells out of the dissociated cells.

Induction of ferroptosis was evident in our iNIL‐MNs upon the addition of RSL3, a small molecule inhibitor of GPx4, to the culture. In addition, deletion of the GPx4 gene using the CRISPR technique decreased the viability of iNIL‐MNs; this effect was reversed by Fer‐1, a specific ferroptosis inhibitor. However, erastin, a system x_c_^−^ inhibitor, was not effective in inducing ferroptosis because the motor neurons in our culture did not express Slc7a11, the target protein for erastin. It is known that astrocytes and neurons are using two different mechanisms in incorporating cysteines, a key precursor for synthesizing cellular glutathione [28]. Astrocytes operate functional system x_c_^−^ transporters and supplement their cysteine pool by uptaking cystine, an oxidized form of cysteine, from the extracellular environment. By contrast, neurons lack the system x_c_^−^ transporter but use excitatory amino acid transporters (EAATs) to increase intracellular cysteine levels [29]. The resistance to erastin was also reported in a paper where the authors used a human iPS‐MN model to test the possibility of ferroptosis induction [30]. Interestingly, erastin was not lethal to the human iPS‐MNs even though the cells expressed an abundant amount of SLC7A11 mRNA [30]. It is possible that the human iPS‐MNs might not have been fully matured and remained expressing SLC7A11 mRNA. Even with the expression of SLC7A11 mRNA, the motor neurons can still be resistant to erastin if cysteines are imported into the neuron through EAATs. Given this information, it is likely that the role of erastin‐induced ferroptosis in neuronal tissues may be revealed if erastin is treated to an organoid culture model or a coculture model of astrocytes and neurons.

Despite several indirect pieces of evidence supporting the role of ferroptosis in ALS [6, 22, 23, 31], the expression of mutant SOD1 (G93A) in our model did not show any differential sensitivity toward RSL3 (Fig. 5A), suggesting a lack of involvement of RSL3‐induced ferroptosis. This finding contrasts with a recent report that demonstrated the protective role of GPX4 overexpression in a SOD1G93A ALS mouse model [31]. However, our data do not necessarily conflict with that result because we aimed to monitor a sensitization effect of SOD1G93A upon RSL3 treatment, rather than the rescuing effect of GPx4 overexpression in degenerating SOD1G93A motor neurons. If the expression of SOD1G93A alone was sufficient to induce cell death in our iNIL‐NM model, we could overexpress GPx4 in the iNIL and examine whether iNIL‐MNs were protected from cell death. Although the expression of SOD1G93A was not cytotoxic, iNIL‐MNs with the *SOD1G93A* mutation showed poor processing of misfolded proteins due to declined efficiency of proteasomal activity [32]. Therefore, an alternative test may be determining whether overexpression of GPx4 in our SOD1G93A motor neurons enhances proteasomal activity or improves the cell's handling of proteostatic stress.

As the SOD1 mutation is one of many genetic causes of ALS, testing RSL3 in other stem cell‐based models bearing other ALS mutations, such as C9orf72 or TDP‐43, would be informative in addressing the possible role of ferroptosis in ALS disease. Motor neurons expressing SOD1G93A accumulate stress and damage created by themselves (cell‐autonomous) or by neighboring glial cells such as astrocytes also expressing SOD1G93A (non‐cell‐autonomous). The detrimental effect of the non‐cell‐autonomous mechanism was clearly demonstrated in various ALS models [33, 34, 35]. Therefore, it would also be interesting to evaluate ferroptosis in a non‐cell‐autonomous model of ALS.

An interesting connection between neuroferritinopathy and ferroptosis was reported in another stem cell‐derived neuron model. Cozzi et al. [36] made iPSCs from neuroferritinopathy patients and found that the cytosol of neurons derived from the patients' iPSCs contained higher iron levels than their isogenic controls. Addition of ferrostatin‐1 rescued neurons from cell death during the differentiation of neuroferritinopathy iPSCs, which suggested a functional role of ferroptosis in high iron‐induced stress affecting the neurons. As exemplified in this paper, we will likely see an increasing number of reports about the functional role of ferroptosis in neurodegeneration as more stem cell‐derived neuron models become available for studying the mechanism of neurodegenerative diseases.

## Materials and methods

### Cell culture

iNIL stem cells were generously provided by Dr Esteban Mazzoni (New York University) and maintained in the 2i‐based media (Table S3) containing leukemia inhibitory factor (ESG1106; Millipore), CHIR99021 (NC0664823; Fisher Scientific, Hanover Park, IL, USA), and PD0325901 (NC0759248; Fisher Scientific). To differentiate iNIL cells into motor neurons, cells were lifted using TrypLE (12‐604‐021; Fisher) and feeder media (Table S4), seeded to a suspension culture dish (08‐772‐32; Fisher) in AK media (Table S5) [37], and incubated for 2 days to allow the embryoid body formation. Doxycycline was added to the suspension culture to induce the expression of three transcription factors, neurogenin‐2, islet‐1, and lhx‐3. These three transcription factors drive neuronal specification in the embryoid body. Two days later, cells were dissociated from the embryoid body using a standard approach, and the dissociated cells were resuspended in the motor neuron media cocktail containing neurotrophic factors (GDNF, BDNF, and CNTF; Table S6). The resuspended cells were plated on poly‐l‐ornithine (PLO)‐coated plates and incubated for 2 days for motor neuron maturation. The drugs were treated to the motor neuron culture at this stage. A more detailed protocol with media recipe for creating motor neurons from iNIL stem cell was described previously [32]. The file Appendix S1 contains tables that show all culture reagents with working concentrations.

### Chemicals and antibodies

Erastin (cat. no. E7781), ferrostatin‐1 (Fer‐1; cat. SML0583), and liproxstatin‐1 (Lpx‐1; cat. SML1414) were purchased from Sigma‐Aldrich (Saint Louis, MO, USA). (1S,3R)‐RSL3 (cat. HY‐100218A) was purchased from MedChem Express (Monmouth Junction, NJ, USA). EGTA‐AM (cat. 20401) and BAPTA‐AM (cat. 15551) were from Cayman Chemical (Ann Arbor, MI, USA). Antibodies against MNX1 (Hb9) were from Developmental Studies Hybridoma Band (cat. 81.5C10) (Iowa City, IA, USA), beta3‐tubulin was from Cell Signaling Technology (cat. 5568) (Danvers, MA, USA), and actin was from Santa Cruz Biotechnology (cat. no. sc‐1616‐R) (Dallas, TX, USA). Antibodies against choline acetyltransferase (ChAT) and GPx4 were from Abcam (cat. ab178859 and ab125066, respectively) (Cambridge, MA, USA). Secondary antibodies against mouse IgG and rabbit IgG were from LI‐COR Biotechnology (cat. 925‐68070 and 925‐68071) (Lincoln, NE, USA).

### Induction of ferroptosis in iNIL‐MN cells

Dissociated motor neuron cells were plated onto two PLO‐coated 96‐well plates (cat. 3904; Corning, Teterboro, NJ, USA) at a density of 150 000 cells per well in a volume of 100 μL. These are the assay plates. Two days later, two empty 96‐well polypropylene plates (cat. 3365; Corning) were labeled as ‘high’ and ‘low’ and filled with 100 μL of motor neuron media except column 4 of ‘high’ plate where 200 μL of 4 μm RSL3 was transferred. Then, twofold serial dilution of the RSL3 across columns 5–11 in ‘high’ and columns 2–9 in ‘low’ was carried out by transferring 100 μL of the compound solution to the next columns successively with mixing. iNIL‐MN cells in the assay plates were treated with RSL3 in a twofold dilution series by transferring 100 μL solution from the compound plates. Finally, the assay plates were returned to the culture incubator and maintained for 24 h before starting resazurin viability assay. The final concentration of RSL3 in the assay plate starts from 2 μm, and then, twofold diluted in the next wells accordingly.

### Viability assay using resazurin

After 1 day of RSL3 treatment, resazurin (cat. R7017; Sigma) was added to the assay plate to a final concentration of 0.01%. The plate was incubated for 1 day to allow live cells to reduce resazurin, resulting in red fluorescence development. The fluorescence intensity of each well was determined using Victor 2 plate reader (Perkin Elmer, Melville, NY, USA) with a 544 nm excitation filter and a 590 nm emission filter. Percent viability was calculated from the following formula using fluorescence intensity values:

$$\%\text{viability}=100^{*}\left(\left(X-N\right)/\left(P-N\right)\right)$$

where *X* is the cells with the lethal compound, *N* is the media only (no cells; negative control), and *P* is the cells without the lethal compound (100% survival; positive control).

### Flow cytometry analysis of lipid peroxidation

Dissociated motor neurons were seeded on a six‐well plate and incubated for 2 days. Cells were treated with RSL3 overnight. The next day, BODIPY™581/591 C11 (cat. D3861; Thermo Fisher Scientific, Waltham, MA, USA) was added to each well to the final concentration of 1 μm, and the culture plate was incubated for 20 min at 37 °C. Cells were harvested using a cell scraper and centrifuged to make a cell pellet. The pellet was resuspended and washed with Hanks' balanced salt solution (HBSS; cat. 14025092; Thermo Fisher Scientific) to remove excess BODIPY‐C11 dye. After washing, cells were pelleted again by spinning, and the cell pellet was resuspended in 500 μL of HBSS. Next, the cell suspension was strained through a 40‐μm cell strainer (BD, San Jose, CA, USA), followed by flow cytometry analysis using Guava easyCyte Plus (Millipore, Billerica, MA, USA). BODIPY‐C11 signal, which reflects the lipid peroxide level, was measured using the FL1 channel. Experiments were performed in biological triplicates, and a representative result was shown.

### Immunohistochemistry

Dissociated motor neurons were seeded in a 24‐well plate at 500 000 cells per well density. Two days later, cells were treated with the indicated concentration of a compound overnight. The next day, cells were fixed with 3.7% formaldehyde solution in PBS for 15–30 min followed by washing with HBSS three times. Next, the cell membrane was permeabilized by incubating cells with cold methanol at −20° overnight. The next day, the methanol was removed, and the permeabilized cells were rehydrated with HBSS. After removing HBSS, the microtubule network was probed with antitubb3 antibody (cat. 5568S; Cell Signaling Technology) in Odyssey blocking buffer with 0.1% Tween 20 for 30–60 min at room temperature followed by washing twice in Odyssey blocking buffer. Alexa Fluor 568 antirabbit antibody (cat. A‐11036; ThermoFisher) was used as the secondary antibody to visualize the microtubule network using the 20× objective lens of an epifluorescence microscope.

### Gene expression analysis by RT‐qPCR

Cells were harvested and cellular RNAs were prepared using Qiagen RNeasy kits (Germantown, MD, USA). Two micrograms of total RNA per sample was subsequently used in a reverse transcription reaction using the TaqMan RT Kit priming with Random Hexamers (cat# 4374966; Fisher Scientific). Primers for the qPCR experiment were designed with Primer Express, and the sequence information was provided in the supplemental information file (Table S7). Quantitative PCR was performed on triplicate samples in 96‐well format using SYBR Green qPCR Master Mix (cat# QP01‐01; Bioland Scientific, Paramount, CA, USA) on a Bio‐Rad CFX96 Real‐Time PCR System (Bio‐Rad Laboratories, Hercules, CA, USA). The change in expression of a gene between experimental and control conditions was computed using the ΔΔ*C*_t_ method with 60S ribosomal protein L38 as an internal reference gene.

### RNA‐seq analysis

Dissociated iNIL‐MNs were plated on PLO‐coated 10‐cm culture dishes. Upon indicated times, cells were harvested using a cell scraper, and RNA was purified using the QIAshredder and RNAeasy extraction kits (Qiagen) according to the manufacturer's instructions. The quality of the RNA sample was monitored by Bioanalyzer (Agilent, Santa Clara, CA, USA), and the RNA samples were submitted to Columbia University Genome Center for RNA‐seq analysis. The list of differentially expressed genes was submitted to Qiagen Ingenuity Pathway Analysis to examine biological pathways altered by drug treatment.

### Generation of INF‐GPx4KO cell line

INF cells were infected with lentivirus harboring pLentiCas9‐blast plasmid (#52962; Addgene, Watertown, MA, USA) and cultured in 2i‐based media containing 10 μg·mL^−1^ of blasticidin. After 2 weeks of selection, the survived cells were pooled and stored as aliquots of frozen stocks in a cryo‐tank. We named this cell line as INF‐Cas‐9 cells. CRISPR oligomers targeting mouse GPx4 (forward: 5′‐CACCGCGTGTGCATCGTCACCAACG‐3′, reverse: 5′‐AAACCGTTGGTGACGATGCACACGC‐3′) were designed and cloned into pLentiGuide‐Hygro‐eGFP plasmid (pLHG; #99375; Addgene) to produce pLHG‐GPx4(guide). INF‐Cas‐9 cells were infected with lentivirus harboring pLHG‐GPx4(guide), and GFP‐positive cells were purified using FACSARIA cell sorter (BD Biosciences). As knocking out GPx4 caused growth inhibition of INF stem cells, we added 1 μm Fer‐1 in the culture media to maintain the culture.

### GFP‐GPx4 overexpression cell line

Human GPx4 cDNA [2] was PCR amplified and cloned into a modified pLVX‐puro plasmid (PT4002‐5; Clonetech, Mountain View, CA, USA) to produce pLVX‐GFP‐GPX4, a lentiviral vector that can express GFP‐GPx4 fusion protein in the target cells. Lentivirus was produced using psPAX2 (#12260; Addgene) and pMD2.G (#12259; Addgene) helper plasmids in 293T cells. INF cells were infected with the lentivirus, and the GFP‐positive cells were purified using the FACSARIA cell sorter.

## Conflict of interest

The authors declare no conflict of interest.

## Author contributions

AMM, AK, CAF, DFR, and WSY performed the experiments. WSY designed the experiments, supervised the project, and wrote the manuscript.

## Supporting information


**Table S1.** RNA‐seq analysis of differentially expressed genes upon RSL3 treatment in iNIL‐MNs.

**Table S2.** RNA‐seq analysis of differentially expressed genes upon treating RSL3 in the presence of Fer‐1 in iNIL‐MNs.

**Appendix S1.** Detailed media information.
**Table S3.** 2i‐based media.
**Table S4.** Feeder media.
**Table S5.** AK media.
**Table S6.** Motor neuron media.
**Table S7.** qPCR primer sequences.

## Data Availability

All the relevant data are within the manuscript and its Supporting Information file. RNA‐seq analysis of differentially expressed genes during RSL3‐induced ferroptosis are provided in the Supporting Information (Tables S1 and S2).

## References

[1] Dixon SJ , Lemberg KM , Lamprecht MR , Skouta R , Zaitsev EM , Gleason CE , et al. Ferroptosis: an iron‐dependent form of nonapoptotic cell death. Cell. 2012;149:1060–72. 22632970 10.1016/j.cell.2012.03.042 PMC3367386

[2] Yang WS , SriRamaratnam R , Welsch ME , Shimada K , Skouta R , Viswanathan VS , et al. Regulation of ferroptotic cancer cell death by GPX4. Cell. 2014;156:317–31. 24439385 10.1016/j.cell.2013.12.010 PMC4076414

[3] Dixon SJ , Patel DN , Welsch M , Skouta R , Lee ED , Hayano M , et al. Pharmacological inhibition of cystine‐glutamate exchange induces endoplasmic reticulum stress and ferroptosis. Elife. 2014;3:e02523. 24844246 10.7554/eLife.02523 PMC4054777

[4] Jiang X , Stockwell BR , Conrad M . Ferroptosis: mechanisms, biology and role in disease. Nat Rev Mol Cell Biol. 2021;22:266–82. 33495651 10.1038/s41580-020-00324-8 PMC8142022

[5] Lewerenz J , Ates G , Methner A , Conrad M , Maher P . Oxytosis/ferroptosis‐(Re‐) emerging roles for oxidative stress‐dependent non‐apoptotic cell death in diseases of the central nervous system. Front Neurosci. 2018;12:214. 29731704 10.3389/fnins.2018.00214 PMC5920049

[6] Chen L , Hambright WS , Na R , Ran Q . Ablation of the ferroptosis inhibitor glutathione peroxidase 4 in neurons results in rapid motor neuron degeneration and paralysis. J Biol Chem. 2015;290:28097–106. 26400084 10.1074/jbc.M115.680090 PMC4653669

[7] Yang WS , Kim KJ , Gaschler MM , Patel M , Shchepinov MS , Stockwell BR . Peroxidation of polyunsaturated fatty acids by lipoxygenases drives ferroptosis. Proc Natl Acad Sci USA. 2016;113:E4966–75. 27506793 10.1073/pnas.1603244113 PMC5003261

[8] Shchepinov MS , Chou VP , Pollock E , Langston JW , Cantor CR , Molinari RJ , et al. Isotopic reinforcement of essential polyunsaturated fatty acids diminishes nigrostriatal degeneration in a mouse model of Parkinson's disease. Toxicol Lett. 2011;207:97–103. 21906664 10.1016/j.toxlet.2011.07.020

[9] Raefsky SM , Furman R , Milne G , Pollock E , Axelsen P , Mattson MP , et al. Deuterated polyunsaturated fatty acids reduce brain lipid peroxidation and hippocampal amyloid beta‐peptide levels, without discernable behavioral effects in an APP/PS1 mutant transgenic mouse model of Alzheimer's disease. Neurobiol Aging. 2018;66:165–76. 29579687 10.1016/j.neurobiolaging.2018.02.024 PMC5924637

[10] Thonhoff JR , Ojeda L , Wu P . Stem cell‐derived motor neurons: applications and challenges in amyotrophic lateral sclerosis. Curr Stem Cell Res Ther. 2009;4:178–99. 19492980 10.2174/157488809789057392 PMC2887342

[11] Mazzoni EO , Mahony S , Closser M , Morrison CA , Nedelec S , Williams DJ , et al. Synergistic binding of transcription factors to cell‐specific enhancers programs motor neuron identity. Nat Neurosci. 2013;16:1219–27. 23872598 10.1038/nn.3467 PMC3820498

[12] Petrov L , Atanassova M , Alexandrova A . Comparative study of the antioxidant activity of some thiol‐containing substances. Cent Eur J Med. 2012;7:269–73.

[13] Skouta R , Dixon SJ , Wang J , Dunn DE , Orman M , Shimada K , et al. Ferrostatins inhibit oxidative lipid damage and cell death in diverse disease models. J Am Chem Soc. 2014;136:4551–6. 24592866 10.1021/ja411006a PMC3985476

[14] Friedmann Angeli JP , Schneider M , Proneth B , Tyurina YY , Tyurin VA , Hammond VJ , et al. Inactivation of the ferroptosis regulator Gpx4 triggers acute renal failure in mice. Nat Cell Biol. 2014;16:1180–91. 25402683 10.1038/ncb3064 PMC4894846

[15] Shimada K , Skouta R , Kaplan A , Yang WS , Hayano M , Dixon SJ , et al. Global survey of cell death mechanisms reveals metabolic regulation of ferroptosis. Nat Chem Biol. 2016;12:497–503. 27159577 10.1038/nchembio.2079 PMC4920070

[16] Gaschler MM , Andia AA , Liu H , Csuka JM , Hurlocker B , Vaiana CA , et al. FINO2 initiates ferroptosis through GPX4 inactivation and iron oxidation. Nat Chem Biol. 2018;14:507–15. 29610484 10.1038/s41589-018-0031-6 PMC5899674

[17] Martinez AM , Mirkovic J , Stanisz ZA , Patwari FS , Yang WS . NSC‐34 motor neuron‐like cells are sensitized to ferroptosis upon differentiation. FEBS Open Bio. 2019;9:582–93. 10.1002/2211-5463.12577 PMC6443867 30984534

[18] Cashman NR , Durham HD , Blusztajn JK , Oda K , Tabira T , Shaw IT , et al. Neuroblastoma x spinal cord (NSC) hybrid cell lines resemble developing motor neurons. Dev Dyn. 1992;194:209–21. 1467557 10.1002/aja.1001940306

[19] Moosmayer D , Hilpmann A , Hoffmann J , Schnirch L , Zimmermann K , Badock V , et al. Crystal structures of the selenoprotein glutathione peroxidase 4 in its apo form and in complex with the covalently bound inhibitor ML162. Acta Crystallogr D Struct Biol. 2021;77:237–48. 33559612 10.1107/S2059798320016125 PMC7869902

[20] Zhivotovsky B , Orrenius S . Calcium and cell death mechanisms: a perspective from the cell death community. Cell Calcium. 2011;50:211–21. 21459443 10.1016/j.ceca.2011.03.003

[21] Xin S , Mueller C , Pfeiffer S , Kraft VAN , Merl‐Pham J , Bao X , et al. MS4A15 drives ferroptosis resistance through calcium‐restricted lipid remodeling. Cell Death Differ. 2022;29:670–86. 34663908 10.1038/s41418-021-00883-z PMC8901757

[22] Shibata N , Nagai R , Uchida K , Horiuchi S , Yamada S , Hirano A , et al. Morphological evidence for lipid peroxidation and protein glycoxidation in spinal cords from sporadic amyotrophic lateral sclerosis patients. Brain Res. 2001;917:97–104. 11602233 10.1016/s0006-8993(01)02926-2

[23] Simpson EP , Henry YK , Henkel JS , Smith RG , Appel SH . Increased lipid peroxidation in sera of ALS patients: a potential biomarker of disease burden. Neurology. 2004;62:1758–65. 15159474 10.1212/wnl.62.10.1758

[24] Rojas F , Gonzalez D , Cortes N , Ampuero E , HernÃ¡ndez DE , Fritz E , et al. Reactive oxygen species trigger motoneuron death in non‐cell‐autonomous models of ALS through activation of c‐Abl signaling. Front Cell Neurosci. 2015;9:203. 26106294 10.3389/fncel.2015.00203 PMC4460879

[25] Watanabe T , Tahara M , Todo S . The novel antioxidant edaravone: from bench to bedside. Cardiovasc Ther. 2008;26:101–14. 18485133 10.1111/j.1527-3466.2008.00041.x

[26] Wichterle H , Lieberam I , Porter JA , Jessell TM . Directed differentiation of embryonic stem cells into motor neurons. Cell. 2002;110:385–97. 12176325 10.1016/s0092-8674(02)00835-8

[27] Kiris E , Nuss JE , Burnett JC , Kota KP , Koh DC , Wanner LM , et al. Embryonic stem cell‐derived motoneurons provide a highly sensitive cell culture model for botulinum neurotoxin studies, with implications for high‐throughput drug discovery. Stem Cell Res. 2011;6:195–205. 21353660 10.1016/j.scr.2011.01.002 PMC3081902

[28] Johnson WM , Wilson‐Delfosse AL , Mieyal JJ . Dysregulation of glutathione homeostasis in neurodegenerative diseases. Nutrients. 2012;4:1399–440. 23201762 10.3390/nu4101399 PMC3497002

[29] Aoyama K , Watabe M , Nakaki T . Modulation of neuronal glutathione synthesis by EAAC1 and its interacting protein GTRAP3‐18. Amino Acids. 2012;42:163–9. 21373771 10.1007/s00726-011-0861-y

[30] Matsuo T , Adachi‐Tominari K , Sano O , Kamei T , Nogami M , Ogi K , et al. Involvement of ferroptosis in human motor neuron cell death. Biochem Biophys Res Commun. 2021;566:24–9. 34111668 10.1016/j.bbrc.2021.05.095

[31] Chen L , Na R , Danae McLane K , Thompson CS , Gao J , Wang X , et al. Overexpression of ferroptosis defense enzyme Gpx4 retards motor neuron disease of SOD1G93A mice. Sci Rep. 2021;11:12890. 34145375 10.1038/s41598-021-92369-8 PMC8213805

[32] An D , Fujiki R , Iannitelli DE , Smerdon JW , Maity S , Rose MF , et al. Stem cell‐derived cranial and spinal motor neurons reveal proteostatic differences between ALS resistant and sensitive motor neurons. Elife. 2019;8:e44423. 31157617 10.7554/eLife.44423 PMC6594754

[33] Ilieva H , Polymenidou M , Cleveland DW . Non‐cell autonomous toxicity in neurodegenerative disorders: ALS and beyond. J Cell Biol. 2009;187:761–72. 19951898 10.1083/jcb.200908164 PMC2806318

[34] Nagai M , Re DB , Nagata T , Chalazonitis A , Jessell TM , Wichterle H , et al. Astrocytes expressing ALS‐linked mutated SOD1 release factors selectively toxic to motor neurons. Nat Neurosci. 2007;10:615–22. 17435755 10.1038/nn1876 PMC3799799

[35] Di Giorgio FP , Carrasco MA , Siao MC , Maniatis T , Eggan K . Non‐cell autonomous effect of glia on motor neurons in an embryonic stem cell‐based ALS model. Nat Neurosci. 2007;10:608–14. 17435754 10.1038/nn1885 PMC3139463

[36] Cozzi A , Orellana DI , Santambrogio P , Rubio A , Cancellieri C , Giannelli S , et al. Stem cell modeling of Neuroferritinopathy reveals iron as a determinant of senescence and ferroptosis during neuronal aging. Stem Cell Rep. 2019;13:832–46. 10.1016/j.stemcr.2019.09.002 PMC6893074 31587993

[37] Wichterle H , Peljto M . Differentiation of mouse embryonic stem cells to spinal motor neurons. Curr Protoc Stem Cell Biol. 2008; Chapter 1: Unit 1H.1.1–9. 10.1002/9780470151808.sc01h01s5 18770634

