# Supplementary material for: Mouse embryonic stem cell‐derived motor neurons are susceptible to ferroptosis
Source: FEBS Open Bio. 2023 Jan 24;13(3):419–33. doi: 10.1002/2211-5463.13545 (PMC9989922; doi:10.1002/2211-5463.13545)
Supplement: Supplementary file 3 — Appendix S1. Detailed media information. Table S3. 2i‐based media. Table S4. Feeder media. Table S5. AK media. Table S6. Motor neuron media. Table S7. qPCR primer sequences. [file FEB4-13-419-s001.docx]

Detailed media information

Table S3. 2i-based media

Table S4. Feeder media

Table S5. AK media

Table S6. Motor neuron media

Table S7. qPCR primer sequences

| RPL32_F | TTCCTGGTCCACAACGTCAAG |
| --- | --- |
| RPL32_R | TGTGAGCGATCTCGGCAC |
| Oct4_F | CAGCAGATCACTCACATCGCCA |
| Oct4_R | GCCTCATACTCTTCTCGTTGGG |
| Mnx1_F | CAGCACCTTCCAACTGGACCAG |
| Mnx1_R | TTCGGCACTTCCCCAAGAGGTT |
| Map2_F | GCTGTAGCAGTCCTGAAAGGTG |
| Map2_R | CTTCCTCCACTGTGGCTGTTTG |
| Olig2_F | ATGCACGACCTCAACATCGCCA |
| Olig2_R | ACCAGTCGCTTCATCTCCTCCA |
| Ki67_F | GAGGAGAAACGCCAACCAAGAG |
| Ki67_R | TTTGTCCTCGGTGGCGTTATCC |
